# Proso Millet (*Panicum miliaceum* L.) and Its Potential for Cultivation in the Pacific Northwest, U.S.: A Review

**DOI:** 10.3389/fpls.2016.01961

**Published:** 2017-01-09

**Authors:** Cedric Habiyaremye, Janet B. Matanguihan, Jade D’Alpoim Guedes, Girish M. Ganjyal, Michael R. Whiteman, Kimberlee K. Kidwell, Kevin M. Murphy

**Affiliations:** ^1^Sustainable Seed Systems Lab, Department of Crop and Soil Sciences, College of Agricultural, Human, and Natural Resource Sciences, Washington State UniversityPullman, WA, USA; ^2^Department of Anthropology, Washington State UniversityPullman, WA, USA; ^3^Food Processing Lab, School of Food Science, College of Agricultural, Human, and Natural Resource Sciences, Washington State UniversityPullman, WA, USA; ^4^International Programs, International Research and Agricultural Development, Washington State UniversityPullman, WA, USA; ^5^College of Agricultural, Consumer, and Environmental Sciences, University of IllinoisUrbana, IL, USA

**Keywords:** proso millet, Pacific Northwest, alternative crops, diversification, nutrition and health benefits, genetics

## Abstract

Proso millet *(Panicum miliaceum* L.*)* is a warm season grass with a growing season of 60–100 days. It is a highly nutritious cereal grain used for human consumption, bird seed, and/or ethanol production. Unique characteristics, such as drought and heat tolerance, make proso millet a promising alternative cash crop for the Pacific Northwest (PNW) region of the United States. Development of proso millet varieties adapted to dryland farming regions of the PNW could give growers a much-needed option for diversifying their predominantly wheat-based cropping systems. In this review, the agronomic characteristics of proso millet are discussed, with emphasis on growth habits and environmental requirements, place in prevailing crop rotations in the PNW, and nutritional and health benefits. The genetics of proso millet and the genomic resources available for breeding adapted varieties are also discussed. Last, challenges and opportunities of proso millet cultivation in the PNW are explored, including the potential for entering novel and regional markets.

## Introduction

### Worldwide Significance of Millets

**FIGURE 1:**
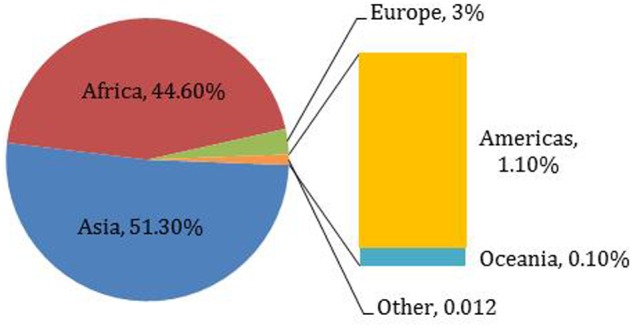
**Worldwide millet production by region, 2014**.

Millets are small-seeded annual cereals grown for food, feed, forage, and fuel (Rachie, 1975; Kothari et al., 2005). About 20 different species of millet have been cultivated throughout the world at different points in time (Fuller, 2006). Commonly cultivated millet species include proso millet *(Panicum miliaceum* L.*)*, pearl millet *(Pennisetum glaucum* L.R. Br.), finger millet *(Eleusine coracana)*, kodo millet *(Paspalum setaceum)*, foxtail millet *(Setaria italica* L. Beauv.), little millet *(Panicum sumatrense)*, and barnyard millet *(Echinochloa utilis)* (Rachie, 1975; Bouis, 2000; Wen et al., 2014). Millet ranks sixth among the world’s most important cereal grains, sustaining more than one-third of the world’s population (Verma and Patel, 2012; Changmei and Dorothy, 2014). Asian and African countries are the biggest millet producers (**Table 1**; **Figure 1**). Millets are a major source of energy and protein for millions of people in China, Japan, Africa, and India, and especially for people living in hot and dry areas of the world (Rachie, 1975; Amadou et al., 2013).

**Table 1:** Top five millet producers in the world, 2014.

Country	Production [t]
India	11,420,000
Niger	3,321,753
China	1,780,000
Mali	1,715,044
Nigeria	1,384,900

*Source: FAOSTAT, 2014 (http://faostat3.fao.org/browse/Q/QC/E)*

Millets are generally among the most suitable crops for sustaining agriculture and food security on marginal lands with low fertility. Millet crops are grown on marginal lands and under low-input agricultural conditions—situations in which major cereal crops often produce low yields (Amadou et al., 2013). Millet can be productive even under harsh growing conditions, especially in regions such as India and Sub-Saharan and West Africa, where average rainfall is often less than 500 mm and soils are sandy and slightly acidic (Changmei and Dorothy, 2014). Of all the millets cultivated in Africa, 74% are grown in Sub-Saharan and West Africa, accounting for 28% of the world’s production (Changmei and Dorothy, 2014). Drought and lack of irrigation are permanent constraints to agricultural production in many developing countries, and are occasional causes of yield loss in developed countries (Ceccarelli and Grando, 1996). An efficient strategy for producing crops under water-deficient conditions is to grow crops adapted to drought instead of crops that require more water (Seghatoleslami et al., 2008). Since millets are adapted to drought conditions, they can be keystone crops to avert food shortage and famine (Amadou et al., 2013).

### Domestication and Spread of Proso Millet

Proso millet was likely domesticated in China sometime around 10,000 BP. Current archeological theorists believe that proso millet domestication took place around the beginning of the Holocene as global temperatures became warmer and hunter-gatherers were exposed to new plants and environments (Bettinger et al., 2007, 2010a,b). A wild ancestor for proso millet has yet to be identified (Miller et al., 2016); however, weedy forms of millet, which may include a wild progenitor, are found across Eurasia (Zohary et al., 2012). Chromosomal *in situ* hybridization with genomic DNA and phylogenetic data provide evidence of the allotetraploid origin of proso millet, with *Panicum capillare* or a close relative, and *Panicum repens* as ancestors (Hunt et al., 2014).

Although archeologists have yet to agree on the exact timing of millet domestication, they generally agree that domestication likely took place separately in three different centers: (1) Northwest China (Bettinger et al., 2007, 2010a,b), (2) Central China (Lu et al., 2009), and (3) Inner Mongolia (Zhao, 2005). From these centers of domestication, millet spread widely throughout East Asia, including high-altitude areas such as the Tibetan Plateau. By the end of the 2nd millennium BP, the cultivation of proso millet had spread to the rest of Central Eurasia and to Eastern Europe (Miller et al., 2016). However, during the 4th millennium BP, worldwide temperatures became cooler (Marcott et al., 2013), and may have led to difficulties in millet cultivation. Evidence shows major shifts in proso millet farming on the Tibetan Plateau until its cultivation was abandoned in Eastern Tibet (Guedes et al., 2014, 2015a,b; Chen et al., 2015; Guedes, 2015). Later, proso millet was largely replaced by wheat and barley on the Tibetan Plateau; however, it continued to be a popular crop in low-lying plains of northern China well after its introduction (Boivin et al., 2012). Warming temperatures in the Himalayan region today may allow farmers to cultivate millet in this area once more. By the fifth millennium BP, proso millet cultivation appears to have spread to Kazakhstan (Frachetti et al., 2010) and Pakistan (Weber, 1991), but whether this crop was grown in these countries before this time is unclear. Site evidence for several finds of proso millet in these areas dates to as early as 8000-7000 BP (Hunt et al., 2008).

### Growth and Environmental Requirements

Proso millet is a summer annual grass, most frequently grown as a late-seeded summer crop (Rao, 1989; Baltensperger, 2002; Williams et al., 2007), and can complete its life cycle within 60–100 days (Baltensperger, 2002). A compact panicle droops at the top like an old broom, hence proso millet’s common name, “broom corn” (Changmei and Dorothy, 2014). Grains are round, about 3 mm long and 2 mm wide, and enclosed in a smooth hull, which is typically white or creamy-white, yellow, or red in color, but may be gray, brown, or black. White-seeded varieties are most often grown in the U.S., followed by red-seeded varieties (Hardman, 1990; Baltensperger, 2002; Williams et al., 2007; Changmei and Dorothy, 2014). Proso millet ranges from 30 to 100 cm tall, with few tillers and an adventitious root system (Baltensperger, 2002).

**FIGURE 2:**
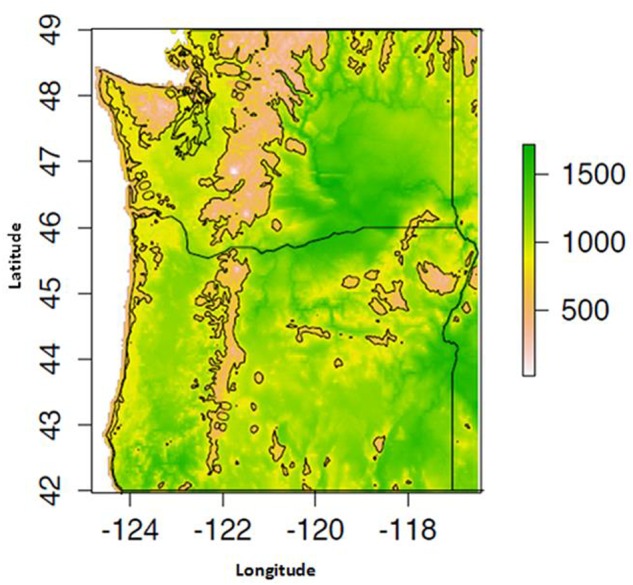
**Mean May–September growing degree day (GDD) availability for the years 1981–2014, computed using 800 m monthly PRISM dataset**.

Proso millet grows further north than the other millets (up to 54°N latitude) and is well adapted to plateau and high-elevation conditions. For example, the plant is found up to 1200 m in the mountains of the former Union of Soviet Socialist Republics (USSR) and up to 3500 m in India (Baltensperger, 2002). Proso millet also grows under non-irrigated conditions in arid lands with as little as 200–500 mm of average annual precipitation (Ceccarelli and Grando, 1996), and can produce grain with only 330–350 mm of annual rainfall (Oelke et al., 1990; Lyon et al., 2008a). As a warm season crop, proso millet is sensitive to frost and requires warm temperatures for germination and development. Optimal soil temperatures for seed germination range from 20 to 30°C (Baltensperger et al., 1995a; McDonald and Copeland, 1997; Amadou et al., 2013). As a C_4_ crop with a low transpiration ratio, proso millet can efficiently fix carbon under conditions of drought, high temperatures, and limited nitrogen and CO_2_. Proso millet avoids drought sensitivity by reaching maturity rapidly (Baltensperger et al., 1995a). In addition, at temperatures above 30°C, proso millet stops vegetative growth, ceases to flower, and maintains its primary stem at a shorter height to better resist drought conditions (Herdrich, 2001; Sateesh, 2010; Changmei and Dorothy, 2014) (**Figure 2**).

Proso millet can be grown on sandy loam, slightly acidic, saline, and low-fertility soils (Riley et al., 1989; Changmei and Dorothy, 2014). However, this crop grows poorly on waterlogged soils (Seghatoleslami et al., 2008; Hunt et al., 2011) and on coarse, sandy soils (Hardman, 1990; Williams et al., 2007). Proso millet thrives in low pH soils and most of its seeds germinate well on soils with pH of 5.5 to 6.5 (Baltensperger et al., 1995a; Lyon et al., 2008b). However, plants grown on soils with pH above 7.8 show symptoms of iron chlorosis. The Palouse region of Washington State in the U.S. has predominantly acidic soils with pH ≤ 5 (Koenig et al., 2011; McFarland et al., 2015), which could seriously constrain millet cultivation.

## Current and Potential Production of Proso Millet in the U.S.

Economically important species of Paniceae (the largest tribe of the Poaceae (Gramineae) grown in the U.S. include proso millet, foxtail millet, and pearl millet (Baltensperger, 2002). Proso millet was first introduced to the U.S. in 1875 by German–Russian immigrants who planted the crop along the eastern Atlantic Coast. Currently, the total cultivated area of proso millet in the U.S. is approximately 204,366 ha (USDA-NASS, 2016). Most of this production is in the semi-arid regions of the Great Plains, where approximately 184,131 ha per year are planted (McDonald and Copeland, 1997; Rajput et al., 2014; USDA-NASS, 2016). The Great Plains region is an area of widespread dryland crop production, with wheat being the dominant crop. Proso millet is among the preferred crops for planting after wheat in the Central Great Plains because it helps control weeds and conserve stored moisture (Anderson, 1990; Lyon et al., 2008b; Krishna, 2013). In 2015, the Central Great Plains states of Colorado, Nebraska, and South Dakota were the major producers of U.S. proso millet, with 109,265 ha, 42,492 ha, and 28,328 ha, respectively (USDA-NASS, 2016). Other states producing significant quantities of proso millet include Kansas, Wyoming, Minnesota, and North Dakota (McDonald et al., 2003).

In the U.S., most of the proso millet crop is utilized for birdfeed and in cattle-fattening rations (McDonald et al., 2003; AGMRC, 2012^1^). However, millet has high nutritional content and is a major source of energy and protein for African and Asian countries (Rachie, 1975; Amadou et al., 2013). Efforts to develop higher yielding and better adapted cultivars could increase the importance of proso millet in the U.S. food and feed industry (Baltensperger et al., 1995a; Berglund, 2007). Among the millet species produced worldwide, proso millet is the most important species traded in the world market, and the U.S. is among the top producers (Castro, 2013). The U.S. generally exports 15–20% of its annual millet production to over 70 countries, primarily as feed. The largest export markets for U.S. proso millet include the Netherlands, the United Kingdom, Canada, and Japan (Powell et al., undated^2^; USDA FAS, 2012). Argentina is a major competitor in this export market (Powell et al., undated)^2^.

## Proso Millet Production in the Pacific Northwest

Historically, millets and other warm season crops such as sudan grass and sorghum were grown as forage and grain feed for livestock and birds in different regions of Oregon (Schoth and Rampton, 1939). In many regions of the PNW, these crops were also grown as temporary or emergency crops in locations or during seasons unfavorable for other, more profitable crops. However, challenges in the marketplace caused a decline in acreage planted to these crops, especially proso millet. Since market opportunities were limited, growers needed to arrange for buyers before planting (Schoth and Rampton, 1939). Moreover, proso millet is associated with birdfeed; hence, concerted marketing is required to change consumers’ perception. Only recently has proso millet been promoted as a whole grain alternative in a healthy diet. Currently, Shepherd’s Grain Cooperative is working with farmers in Idaho and Washington to grow proso millet and other warm season crops, including sunflower, teff, amaranth, and sorghum. From 2014 to 2016, based on interviews conducted during the Shepherd’s Grain field days, growers expressed satisfaction about the inclusion of proso millet in their crop rotations (Jeremy Bunch and Eric Odberg, personal communication).

According to Herdrich (2001), of all the millets, proso millet would perform best in the PNW, largely because it can be grown as a dryland crop, without supplemental irrigation. Almost all proso millet produced in the U.S. is grown under conventional dryland/rainfed conditions in Colorado, Nebraska, and South Dakota (Schaible and Aillery, 2012). Only a small area in these states is irrigated (McDonald et al., 2003). Of the approximate 4,046 irrigated hectares of proso millet in the U.S., about one-half are located in Nebraska (Schaible and Aillery, 2012). Proso millet is only irrigated for specific reasons, such as when it is planted as a replacement for irrigated wheat lost due to late hail damage. Nebraska has been the second highest producer of proso millet in the U.S.; in 2012, proso millet production contributed $13 million to the state’s economy (Santra, 2013).

Growers in the PNW could also benefit from proso millet’s ability to produce grain under limited water conditions on marginal soils, with minimal agronomic inputs (Oelke et al., 1990; Santra, 2013). Although proso millet can be grown on various soil types and climate conditions, it thrives on well-drained loamy soils (Baltensperger, 2002). This soil type is predominant throughout the Columbia Plateau and across the intermediate precipitation zone of the inland dryland areas, including eastern and central Washington, eastern and north-central Oregon, the Idaho panhandle, and the intermountain region of southern Idaho (Schillinger et al., 2003; Williams et al., 2014). Another benefit of growing proso millet is that it can be grown as a catch crop when other crops fail or planting is delayed due to unfavorable weather (Hardman, 1990; Oelke et al., 1990; Herdrich, 2001; Adekunle et al., 2016).

## Crop Rotation

The planting time of proso millet fits well in rotation with winter annual crops such as winter wheat or warm-season broadleaf crops such as sunflower (Herdrich, 2001). Successful proso millet production in Nebraska is attributed to the practice of eco-fallow—planting proso millet in standing wheat stubble in the spring to control weeds and to conserve stored soil moisture (Anderson, 1990). Potential problems for millet production in the PNW include competition from grassy weeds, summer annual broadleaf weeds, and perennial broadleaf weeds (Herdrich, 2001). Baltensperger et al. (1995a) found that planting millet after sunflowers provides more options for broadleaf weed control. Because proso millet has a shallow root system, it is often planted after sunflowers that have deep, extensive root systems and often deplete soil water at 6 feet or deeper. This deep depletion of soil water restricts the option of planting another deep-rooted crop after sunflowers, unless summer fallow is used to help restore soil water (Lyon et al., 2008b). A winter wheat/sunflower/proso millet/fallow rotation has been successful for some growers in the western Great Plains (Baltensperger et al., 1995a).

Proso millet can also be used in rotation with corn or sorghum because it tolerates atrazine, a primary chemical input in corn and sorghum production systems. The warmer soil temperatures in corn or sorghum stubble fields toward the end of spring due to high plant biomass, allow proso millet to be planted earlier (Lyon et al., 2008b). The density of the summer annual weed seedbank can decline by nearly 90% if the proso millet crop is followed by two winter crops or by a winter crop and fallow period (Anderson et al., 1999). An earlier study found that grain yield of proso millet increased 23% when fall weeds were reduced by sweep plowing after wheat harvest (Anderson et al., 1986). More recent studies have found that adding proso millet into a winter wheat/fallow rotation can extend and diversify this crop rotation system to provide multiple benefits. These benefits include helping to: (1) control winter annual grass weeds, (2) manage disease and insect pressure, and (3) preserve deep soil moisture for wheat (Lyon et al., 2008b; Santra, 2013).

In low-rainfall environments of the PNW, the traditional, 2-year winter wheat/summer fallow rotation is the most economical production system. This crop rotation is commonly employed in regions such as the Columbia Plateau that receives less than 330 mm of annual rainfall, though it is considered inadequate to produce crops every year (Rasmussen et al., 1994, 1998). In the Columbia Plateau, the winter wheat/summer fallow rotation is employed by growers to store winter precipitation and control weeds (Rasmussen et al., 1998). In contrast, in areas which receive higher annual rainfall (375–550 mm), growers use 2- to 5-year rotation systems that include winter wheat-spring barley or spring wheat/summer fallow (Rasmussen et al., 1998; McCool and Roe, 2005).

Annual cropping in the PNW is limited by low rainfall and soil moisture. According to Rasmussen et al. (1998) and Williams et al. (2014), annual cropping that includes alternative crops and spring planting has better weed and disease management compared to summer fallow. In addition, Rasmussen et al. (1998) recommended using an annual cropping system to maintain soil organic matter. In their study, Rasmussen et al. (1998) found that any cropping system with summer fallow lost soil organic matter due to high biological oxidation and absence of C inputs during the fallow year. Warm season crops such proso millet could replace summer fallow in winter wheat-fallow rotations. Growing crops such as proso millet instead of summer fallow would provide more surface cover and help growers meet conservation practice requirements. As an added benefit, proso millet can be an extra cash crop in a wheat-proso-fallow rotation. Furthermore, proso millet can be a catch crop to compensate for wheat crop loss due to freezing, wind erosion, drought, or hail (Baltensperger, 1996).

**FIGURE 3:**
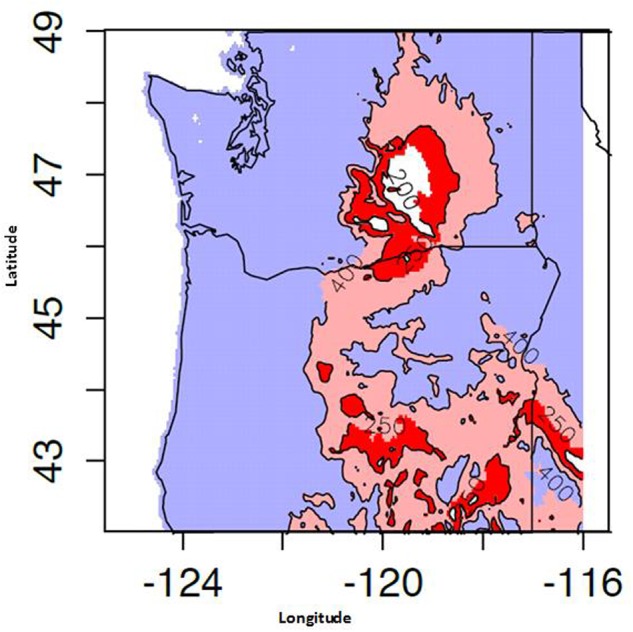
**Mean May–September precipitation availability for the years 1981–2014. Computed using 800 m monthly PRISM dataset**.

Proso millet is one of the most efficient crops for removing water from the topsoil and converting it to dry matter, because its root depth is generally limited to the upper 92 cm of soil. Additional summer moisture is beneficial, as it can replenish the low water reservoir in the subsoil for proso millet’s use (Baltensperger et al., 1995a). However, growers in some regions of PNW are not guaranteed additional summer moisture due to the region’s typical weather patterns. The PNW has hot, dry summers, and most rainfall comes during the winter months, with average annual precipitation below 330 mm (**Figure 3**). This weather pattern includes areas east of the Cascade Mountains where average annual rainfall is much less than 330 mm. Some farming regions in this environment receive as little as 180 mm (CIG)^3^. The Palouse region of Washington only receives, on average, approximately 60 mm of summer precipitation (WeatherDB^4^). This low summer moisture might pose a challenge to growing proso millet in the PNW, especially if precipitation does not occur when most needed. The first 2 weeks are critical when growing proso millet. For example, lack of rain during the first 2 weeks after planting can cause a poor stand and subsequent yield loss (Baltensperger et al., 1995a). On the other hand, if sufficient rainfall occurs later in the season, proso millet can still produce a reasonable yield despite a long, early period of limited rainfall (McDonald et al., 2003).

In Nebraska, the grain yields of proso millet respond more consistently to soil water at planting than do other long-duration crops such as corn, grain sorghum, or sunflower (Baltensperger et al., 1995a; Lyon et al., 2008b). This finding suggests that soil water levels at planting may be a reliable predictor of grain yields (Baltensperger et al., 1995a). In a study comparing conventional tillage, reduced tillage, and no-tillage production systems, results indicated that wheat-corn-proso millet and wheat-millet rotations produced almost double the total grain yield compared to a wheat-fallow rotation (Anderson et al., 1999). Continuous cropping with a wheat-corn-proso millet rotation using no-till management would increase the soil concentration of glomalin, which is an important molecule in aggregate stabilization (Wright and Anderson, 2000). Anderson et al. (1999) and Nielsen et al. (2005) stated that producers could grow crops more frequently if crop residues are maintained on the soils through reduced tillage or no-till. Soil-water relations change under no-till systems, with higher water content in the 0–15 cm soil layer and less evaporation from the soil surface due to the presence of continuous crop residues (Anderson, 1990).

Often, growers who wish to avoid summer fallow plant proso millet as a transition crop when rotating back to winter wheat from a full-season summer crop (Baltensperger et al., 1995a). Since proso millet has a shallow root system, the top 92 cm of the soil profile is quite dry after harvest, but the deeper soil water has been preserved for use by winter wheat plants in the spring (Baltensperger et al., 1995a; McDonald et al., 2003). Moreover, winter wheat sown into proso millet stubble under no-till systems are less prone to damage from blowing soil compared to those planted into summer fallow. Increasing soil residue levels may also improve snow capture, providing much needed moisture for the crop (Anderson, 1990; Baltensperger et al., 1995a).

## Nutritional and Health Benefits of Proso Millet

Millets are a major source of energy and protein and have high nutritive value, comparable to major cereals such as wheat, rice, and maize (Amadou et al., 2013; Saleh et al., 2013) (**Table 2**). Millets are unique among the cereals because of their high calcium, iron, potassium, magnesium, phosphorous, zinc, dietary fiber, polyphenols, and protein content (Hulse et al., 1980; Devi et al., 2014; Gupta et al., 2014). Millets are gluten-free, ideal for people who are gluten-intolerant, though millet flour cannot be used for raised bread (Hulse et al., 1980; Thompson, 2009; Amadou et al., 2013; Santra, 2013). Millets are easy to digest. They contain a high amount of lecithin, which provides excellent support for nervous system health by helping to restore nerve cell function, regenerate myelin fiber, and intensify brain cell metabolism. Millets are also rich in micronutrients such as niacin, B-complex vitamins, Vitamin B6, and folic acid (Hulse et al., 1980; Pathak, 2013). Millets generally contain significant amounts of essential amino acids, particularly those containing sulfur (methionine and cysteine). Saleh et al. (2013) reported that millets are good sources of essential amino acids, except lysine and threonine, but are relatively high in methionine. Millets also have higher fat content than maize, rice, and sorghum (Obilana and Manyasa, 2002).

**Table 2:** Nutritional composition of proso millet (*Panicum miliaceum* L.) compared to other small millets, wheat and rice (100 g).

Crop	Protein (g)	Carbohydate (g)	Fat (g)	Dietary fiber (g)	Mineral matter (g)	Calcium (g)	Phosphorus (mg)	Fe (mg)
Proso millet (*Panicum miliaceum* L.)	12.5^∗^	70.4	3.1	14.2	1.9	14	206	10.0
Finger millet *Eleusine coracana* (L.) Gaertn.	7.3	72.0	1.3	18.8^∗^	2.7	344^∗^	283	3.9
Kodo millet *Paspalum scrobiculatum* L.	8.3	65.0	1.4	15.0	2.6	27	188	12.0
Foxtail millet *Setaria italica* (L.) P. Beauv.	12.3	60.9	4.3	14.0	3.3	31	290	5.0
Little millet *Panicum sumatrense* Roth ex Roem. and Schult.	7.7	67.0	4.7	12.2	1.5	17	220	6.0
Barnyard millet *Echinochloa esculenta* (A. Braun) H. Scholz	6.2	65.5	2.2	13.7	4.4^∗^	11	280	15.0^∗^
Wheat *Triticum aestivum* L.	11.8	71.2	1.5	12.9	1.5	41	306	3.5
Rice *Oryza sativa* L.	6.8	78.2	0.5	5.2	0.6	45	160	1.8

*^∗^Higher than major cereals and wheat, adapted from Saha et al. (2016)*.

Ravindran (1991) reported the composition of the various millets, including the four varieties of foxtail millet (*Setaria italica*). The average protein contents of common millet, finger millet and foxtail millet were 14.4, 9.8 and 15.9%, respectively. The crude fiber content of the millets ranged from 3.2 to 4.7%. In general, the mineral contents were high compared with those of other common cereal grains. Considerable between- and within-millet differences were observed with regard to most nutrients analyzed. The overall results are suggestive of the underexplored potential of millets as sources of dietary nutrients. The protein content in proso millet is around 11% (dry basis) (Kalinova and Moudry, 2006). They reported that the proso millet protein is richer in essential amino acids (leucine, isoleucine, methionine), compared to wheat. Hence, the protein quality of proso (Essential Amino Acid Index) was higher (51%) compared to wheat. Lorenz and Dilsaver (1980) conducted a thorough study on the milling characteristics and proximate composition and nutritive value of the proso-millet flours. Compared with wheat, millet flours were high in ash and crude fat and were higher in protein content.

Millet has many nutritional and medical functions (Obilana and Manyasa, 2002; Yang et al., 2012). Millets are rich in health-promoting phytochemicals and considered functional foods (Pathak, 2013). Consumption of proso millet and other millets is associated with reduced risk of type-2 diabetes mellitus because whole grains like millet are a rich source of magnesium. Magnesium acts as a co-factor in a number of enzymatic reactions that regulate the secretion of glucose and insulin. Magnesium can also reduce the frequency of migraine headaches and heart attacks, and is beneficial for people suffering from atherosclerosis and diabetic heart disease (Shobana and Malleshi, 2007; Gélinas et al., 2008). Millet is a good source of phosphorus, which plays a vital role in maintaining cell structure in the human body (Kumari and Sumathi, 2002). Phosphorus in millet helps in the formation of the mineral matrix of the bone and is an essential component of ATP, which is the energy currency of the body (Kumari and Sumathi, 2002; Liang et al., 2010; Devi et al., 2014). A single cup of cooked millet provides around 24% of the body’s daily phosphorus requirement. Furthermore, phosphorus is a very important constituent of nucleic acids, which are the building blocks of the genetic code (Kumari and Sumathi, 2002).

Since millets are high in fiber, antioxidants, and complex carbohydrates, they can be valuable in preventing CVD and cancer. Coulibaly et al. (2011) stated that millet is an important crop with a wide range of health benefits due to the phytochemicals in millet grains. Zhang et al. (2014) studied the phytochemical content, antioxidant activity, and anti-proliferative properties of three diverse varieties of proso millet. Anti-proliferative activities were also studied *in vitro* against MDA human breast cancer and HepG_2_ human liver cancer cells. Results showed a differential and possible selective anti-proliferative property of the proso millet. Consuming millets can lower cholesterol and phytate, which are associated with CVD and cancer risk. Lignans, essential phytonutrients present in millet, act against different types of hormone-dependent cancers, such as breast cancer, and help reduce the risk of heart disease (Coulibaly et al., 2011). Regular consumption of millet can reduce high blood pressure and high cholesterol levels in postmenopausal women suffering from signs of CVD (Shahidi and Chandrasekara, 2013). Millets can also slow down the aging process in humans (Pathak, 2013; Shahidi and Chandrasekara, 2013) and may protect against age-onset degenerative diseases (Pathak, 2013).

Millet protein has a beneficial influence on metabolism of cholesterol (Nishizawa and Fudamo, 1995). Nishizawa et al. (2002) examined the effects of dietary proso-millet protein on plasma levels of high-density lipoprotein (HDL) cholesterol in different rats from animals reported in our previous studies. They reported that the ingestion of the millet protein elevates plasma levels of HDL-cholesterol like our earlier works. Taking into account the anti-atherogenic function of HDL, therefore, the millet protein would be useful as a new food ingredient, which has the function that regulates cholesterol metabolism. This protein is also considered beneficial in the prevention of liver injury (Nishizawa et al., 2002).

Millet can be cooked and prepared in different ways. The grains can be boiled, steamed to make salad, or fully cooked similar to rice. Together with other ingredients, millet flour can be made into porridge (Santra, 2013). Because of its mild flavor, light color, gluten-free quality, and potential health benefits, proso millet has been receiving growing interest from the food industries in Europe and North America (Santra, 2013). Organic proso millet has a niche market because of its nutritional properties (UNL NebGuide Alternative uses of Proso Millet, G2218)^5^. In addition, the market for gluten-free food in the U.S. bread and grain industry is growing. This market grew 17% in 2012 and is projected to be worth $6.6 billion by 2017 (CDA, 2016^6^). Furthermore, proso millet can be a substrate in distilled liquors and beers and is used to make fermented beverages in Africa and Asia. Thus, proso millet may also gain traction in the U.S. and European alcohol markets, especially in the gluten-free sector (Santra et al., 2015).

There is scarce information about millet integration and adoption into the food industry (Saleh et al., 2013). According to the Common Fund for Commodities (CFC) and International Crops Research Institute for Semi-Arid Tropics (ICRISAT) (CFC and ICRISAT, 2004), the industrial application of millets in developing countries is facing increasing competition from other industrially produced grain crops. Even though there are some studies reported in the literature about the nutritional composition, health benefits and uses of proso millet, there is still a broad gap in the literature specifically on the nutritional composition of different varieties and its applications in different food products. Studies on the processing methods to make diverse food products from proso millet are necessary. Information generated from these studies should be available to stakeholders in the food processing industry so that proso millet can be included as an ingredient in food products. More research is required to breed millet cultivars suited to different agro-ecosystems and to formulate best production practices. There is a need to correlate the agronomic characteristics with the nutritional properties and end-uses of proso millet. Efforts to educate people about the potential value of millets, including nutritional properties and health benefits, would help create market demand (Amadou et al., 2013; Saleh et al., 2013). In addition, conducting market research on proso millet and promoting it as an alternative whole grain in a healthy diet are essential for acceptance and consumption in developed countries (Amadou et al., 2013).

## Genetics and Genomics of Proso Millet

Proso millet is considered a minor crop compared to wheat, barley and potatoes, and this status is reflected not only in the amount of land cultivated but also in the extent of research in its genetics, genomics and breeding (Rajput and Santra, 2016). Kubešová et al. (2010) estimated the haploid genome size of *P. miliaceum* to be 1.04 pg or 1017.2 Mb (1C value). The use of molecular markers, generation of sequence information, creation of mapping populations and mutants, and construction of genetic maps, are prerequisites for genetic studies and molecular plant breeding in any crop (Varshney et al., 2010; Lata, 2015). At present, the genomic resources available for *P. miliaceum* are several types of molecular markers such as simple sequence repeat (SSR) and single nucleotide polymorphism (SNP) markers, expressed sequence tags (ESTs), sequences of the Waxy gene, miRNAS, gene-based markers, a genetic linkage map, and an assembled and characterized transcriptome (**Table 3**).

**Table 3:** List of molecular markers reported in proso millet (*Panicum miliaceum* L.).

DNA markers	Number of markers	Reference
Random amplified polymorphic DNA (RAPD)	Five	Colosi and Schaal, 1997
Amplified fragment length polymorphism (AFLP)	Eight primer pairs which amplified a total of 450 fragments, 339 of which were polymorphic	Karam et al., 2004, 2006
Inter simple sequence repeats (ISSR)	Seven	Lágler et al., 2005
	Eight	Trivedi et al., 2015
Polymerase chain reaction (PCR)-based markers	Six primer pairs for the intron splice junction (ISJ)	Hu et al., 2008
Primers from 5S rDNA repeats	Two types of repeats different in the length of the NTS region	Pak et al., 2012
microRNAs (miRNAs)	43 potential miRNAs which may regulate 68 target genes	Wu et al., 2012
Sequence related amplified polymorphism (SRAP)	40	Trivedi et al., 2015
Simple sequence repeat (SSR)	46 markers from other plant species (21 from rice; 15 from wheat; 9 from oat; 1 from barley)	Hu et al., 2009
	25 markers from proso millet by constructing a SSR-enriched library from genomic DNA	Cho et al., 2010
	348 markers from switchgrass, of which 254 were highly polymorphic in proso millet	Rajput et al., 2014
	11 SSR markers developed from foxtail millet	Kumari et al., 2013; Trivedi et al., 2015
	500 primer pairs developed by high-throughput sequencing; 67 polymorphic SSR primers used in study	Liu et al., 2016
	35,000 loci discovered through transcriptome characterization	Yue et al., 2016a
Gene-specific primers	Three primer pairs based on sequences of the *GBSSI* (*Waxy*) gene reported by Hunt et al., 2010 to distinguish waxy genotypes	Araki et al., 2012
	Four Dof domain and 20 *Dof* genes specific primers; for the coding regions of the Dof (DNA binding with One Finger) transcription factor	Kushwaha et al., 2015
Expressed sequence tags (ESTs)	211 ESTs, all derived from drought stress induced leaf tissues	Saha et al., 2016
Differentially expressed genes	62, 543 unigenes functionally annotated from the *de novo* assembly and characterization of the proso millet transcriptome	Yue et al., 2016a
	32 PmWRKY genes involved in abiotic-stress response	Yue et al., 2016b
Single sequence polymorphism (SNP)	833 SNP markers used to construct a genetic linkage map and conduct QTL-linkage study	Rajput et al., 2016
	406,000 SNP loci identified in the transcriptome of proso millet	Yue et al., 2016a

### Genetic Diversity Studies Using SSR Markers

Conservation and increased use of proso millet germplasm, especially for breeding new cultivars, requires information on its genetic diversity (Hu et al., 2008; Dvořáková et al., 2015). Genebanks worldwide hold a rich collection of proso millet accessions, especially in areas where proso millet is still grown. There are 24,014 proso accessions held in 10 institutions based in China, Russia, India and the U.S. (**Table 4**). Moreover, farmers grow and preserve landraces of proso millet, often in remote areas of the world, thus maintaining its agricultural and functional diversity. These landraces have helped agrarian communities survive for generations in marginal lands (Newmaster et al., 2013). Scientists have examined the genetic diversity of proso millet accessions to investigate the genetic relationships among landraces, breeding lines and cultivars, construct phylogenetic trees and draw connections between genetic diversity and geographical origins. Several types of molecular markers have been used to estimate genetic diversity and relatedness in *P. miliaceum* accessions, including amplified fragment length polymorphism (AFLP) (Karam et al., 2004, 2006), random amplified polymorphic DNA (RAPD) (M’Ribu and Hilu, 1994; Colosi and Schaal, 1997), cleaved amplified polymorphic DNA (CAP) (Lágler et al., 2005), inter simple sequence repeats (ISSR) (Lágler et al., 2005; Trivedi et al., 2015), sequence related amplified polymorphism (SRAP) (Trivedi et al., 2015) and simple sequence repeat (SSR) polymorphic markers (Hu et al., 2009; Cho et al., 2010; Hunt et al., 2010, 2011; Rajput et al., 2014; Dvořáková et al., 2015; Rajput and Santra, 2016). More genetic diversity studies using SSR markers have been conducted compared to other PCR-based markers because SSR markers are (1) neutral, abundant, and evenly distributed in the genome; (2) more informative since they are co-dominant, multi-allelic, and have high polymorphic information (PIC); and (3) easier to reproduce and score (Rajput et al., 2014).

**Table 4:** Germplasm collections of proso millet (*Panicum miliaceum* L.) and institute headquarters.

Country	Number of accessions		Institution
Russian Federation	8,778		N.I. Vavilov All-Russian Scientific Research Institute of Plant Industry
China	6,517		Institute of Crop Germplasm Resources, Chinese Academy of Agricultural Science (ICGR-CAAS)
Ukraine	5,022	3,976	Ustymivka Experimental Station of Plant Production
		1,046	National Centre for Plant Genetic Resources of Ukraine, Yuryev Plant Introduction Institute UAAS
U.S.	1,432	719	Plant Genetic Resources Conservation Unit, USDA-ARS, Griffin, GA, U.S.
		713	North Central Regional Plant Introduction Station, USDA-ARS, Ames, Iowa, U.S.
India	842		International Crops Research Institute for the Semi-Arid Tropics (ICRISAT)
Poland	721		Botanical Garden of the Plant Breeding and Acclimatization Institute in Bydgoszcz
Mexico	400		Estacin de Iguala, Instituto Nacional de InvestigacionesAgricolas (INIA)
Japan	302		National Institute of Agrobiological Sciences (NIAS)

*Adapted from Dwivedi et al. (2012) and Goron and Raizada (2015)*.

Since there are limited genomic resources for proso millet, SSR markers have been derived from available sequence data of other plant species (Hu et al., 2009; Rajput et al., 2014; Rajput and Santra, 2016). Hu et al. (2009) used 46 SSR markers from rice, wheat, oat and barley to examine the genetic diversity of 118 Chinese accessions with different ecotypes. The genetic similarity (GS) coefficients among the accessions were moderate to high and Hu et al. (2009) grouped the accessions into five clusters which closely corresponded with the ecological areas of the collection sites. The clustering of accessions was also consistent with the GS matrix. Rajput et al. (2014) used comparative genomics to develop SSR markers from sequences of switchgrass (*Panicum virgatum* L.). Since switchgrass is taxonomically the closest species to proso millet, 62% of the markers they tested on eight genotypes were amplified. Of these, 254 were highly polymorphic and generated 984 alleles. Interestingly, eight of these polymorphic markers correspond to highly conserved sequences in plants associated with drought and flood tolerance.

Cho et al. (2010) developed the first SSR markers for proso millet by constructing a SSR-enriched library from genomic DNA. They tested 25 markers on 50 accessions of *P. miliaceum* and detected 110 alleles. Hunt et al. (2011) used 16 of the markers developed by Cho et al. (2010) to examine the genetic diversity and phylogeography of 98 landrace accessions across Eurasia. In their study, they found strong geographic structuring where two genetic clusters had marked correspondence with two (eastern and western) geographic clusters. The eastern cluster was further divided into four sub-clusters or gene pools, while the western cluster into two sub-clusters. The eastern cluster included samples from China, Mongolia, Nepal and northeastern India, the Russian Far East, Korea and Japan; while the western cluster included samples from Ukraine, the Caucasus, European Russia, central Asia, northwestern India, Pakistan, and ten samples from China and Mongolia. In their study, Hunt et al. (2011) traced the molecular trail of proso millet through the Eurasian steppe region, a trail generated by genetic variation, evolutionary and population processes, linkage of SSR loci and genes coding for adaptive traits, and selection by humans for culinary traits. Using archeobotanical and molecular diversity data, Hunt et al. (2011) postulated various theories on the spread of proso millet from its centers of domestication.

Using a mix of SSR markers from other species and those developed on proso millet, Rajput and Santra (2016) screened 709 SSR markers on eight diverse genotypes, with the goal of examining the genetic diversity of proso millet germplasm in the U.S. Of these, 100 SSR markers were polymorphic, including 80 from switchgrass (Rajput et al., 2014), six from proso millet (Cho et al., 2010), and 14 from other species – rice (7), wheat (5), oat (2) (Hu et al., 2009). These polymorphic markers were then used on 90 proso millet genotypes representative of the whole USDA-ARS collection based in Ames, Iowa. The 90 genotypes could be considered a core collection since it consists of accessions from 25 countries, including 14 U.S. and one Canadian cultivar. Analysis of morphological and agronomic traits and molecular data revealed a wide range of genetic diversity in the core collection. However, all cultivars developed in the U.S. were grouped into one cluster. All 14 U.S. cultivars were developed through selection of landraces and conventional plant breeding but only six of these cultivars are still cultivated in the U.S. According to Rajput and Santra (2016), the genetic base of these six cultivars is narrow since only a few lines were used as parents. The work of Rajput and Santra (2016) are particularly relevant to the potential inclusion of diverse proso millet accessions in PNW crop rotations. Their results will also help future breeding programs for proso millet in the PNW, especially in the selection of parents for important traits such as tolerance to biotic and abiotic stresses.

Liu et al. (2016) used high-throughput sequencing to develop SSR markers specific to proso millet and thus increase the number of SSR markers researchers can use. They generated 500 primer pairs which they screened on eight accessions randomly selected from a pool of 73 Chinese accessions. Of these, 162 primer pairs produced polymorphic and reproducible fragments. From these primer pairs, 67 SSR markers were developed and used to examine the genetic diversity of 88 accessions consisting of landraces and cultivars. They detected 179 alleles and 349 genotypes, revealing a moderate level of genetic diversity. The 88 accessions were separated into four groups with a GS level of 0.633 by cluster analysis based on UPGMA. The clustering based on genetic diversity also corresponded to geographical regions, similar to the results obtained by M’Ribu and Hilu (1994), Hu et al. (2009), and Hunt et al. (2011). Cultivars were also grouped according to the geographical regions in which they were registered, with specific varieties and their parents often placed in the same group, also similar to the findings of M’Ribu and Hilu (1994), Hu et al. (2009), and Cho et al. (2010). These results suggest that breeding of proso millet in specific regions has proceeded in isolation (Liu et al., 2016). Similar to Trivedi et al. (2015), Liu et al. (2016) observed abundant morphological variation in the Chinese accessions, but more molecular markers or different types of markers are needed to assess their genetic diversity. These Chinese gene pools could also be valuable to future proso millet breeding programs since some accessions and ecotypes could be suitable to particular regions in the PNW.

### PCR-Based Molecular Markers for Genetic Diversity Analysis

Instead of using markers developed from non-coding regions of the genome, primers from the coding region of genes or conserved domains can be used for genetic diversity analysis. Hu et al. (2008) used the polymerase chain reaction (PCR) to examine the genetic diversity of 32 proso millet accessions from China, together with six Indian accessions for comparison. They used primers designed for six intron splice junctions (ISJ) described previously (Weining and Langridge, 1991; Weining and Henry, 1995) together with long random primers to generate 56 DNA fragments, of which 42 (75%) were polymorphic and reproducible. The clustering of accessions largely corresponded with the geographical locations of the origins of the accessions, similar to the results of M’Ribu and Hilu (1994), Hu et al. (2009), and Hunt et al. (2011). The PCR analysis used by Hu et al. (2008) also distinguished between landraces and cultivars. There was also a clear association of the glutinous trait (waxy)/non-glutinous trait (non-waxy) with genetic and geographical clusters.

Hunt et al. (2012) used the mutations in the *Waxy* gene to examine the phylogeny of Eurasian landraces, while Araki et al. (2012) developed markers based on these mutations to conduct the first phylogenetic study of Japanese landraces. The *Waxy* gene encodes glucose bound starch synthase (GBSS) in cereals, and the recessive allele (*wx*) has loss of function of the enzyme. Since proso millet is a tetraploid, the *Waxy* gene has two loci, derived from its diploid ancestors. Hunt et al. (2010) sequenced the *Waxy* gene and reported that the gene has two homeologues, the L-form and the S-form, which have distinct gene sequences and intron lengths. The L-form has three alleles: one wildtype (L_C_) and two mutants resulting from sequence polymorphisms - an insertion of adenine residue causing a frame shift (L_f_) or substitution of cysteine with tyrosine (L_Y_). On the other hand, the S-form has two alleles, the wildtype (S_0_), and a mutant arising from a 15-bp deletion resulting to the loss of five amino acids (S_-15_). There are six possible combinations of these alleles, but only five combinations have been observed in the Eurasian landraces (Hunt et al., 2010). All the waxy accessions in their study had either of these two allele combinations, S_-15_/L_Y_ or S_-15_/L_f_. Examination of the spread of these alleles show distinct spatial distributions and correspond to the phylogeography of proso millet when examined with SSR markers (Hunt et al., 2012). Araki et al. (2012) developed PCR-based markers for the *Waxy* gene and used these markers to conduct the first phylogenetic study of Japanese landraces. They found that the genotype S_-15_/L_Y_ is prevalent among the waxy landraces in Japan. They also hypothesized that the non-waxy genotype (S_0_/L_Y_) was introduced to Japan from northeast China, while the waxy genotype S_-15_/L_Y_ came from Korea. The northern part of East Asia, specifically the Primorskaya Province of Russia, could have also been the source of the other waxy genotype S_-15_/L_f_ as well as another pathway by which the non-waxy genotype came to Japan. These markers would be effective in genotyping proso millet accessions for the waxy/non-waxy trait in the U.S. core collection, and for other proso millet collections in the world.

Pak et al. (2012) designed primers from 5S ribosomal sequences to isolate the complete non-transcribed spacer (NTS) sequence to construct phylogenetic relationships of accessions from Korea, China, and Russia. Most of the genotypes from China and Russia clustered together, while the Korean genotypes clustered in another group. Kushwaha et al. (2015) used primers for the coding regions of the Dof (DNA binding with One Finger) transcription factor to compare the diversity of cereals and millets. The Dof transcription factors belong to a family of zinc-finger transcription factors widely distributed in the Plant Kingdom and involved in the regulation of biological processes exclusive to plants. Since there is a broad variation in the number of *Dof* genes in different crops, these can be employed for diversity analysis between genera and within a genus. Kushwaha et al. (2015) used four Dof domains and primers specific to 20 *Dof* genes. Analysis of the banding patterns generated by 35 sets of Dof domain and gene-specific primers separated the accessions into two major clusters. One cluster was composed of rice, sorghum, maize, finger millet, foxtail millet, barnyard millet and proso millet, while the other cluster was composed of wheat, barley, oat, little millet and kodo millet. Potentially, the DNA bands could be cloned and sequenced and give insight to the proso millet genome through comparative genomics (Kushwaha et al., 2015).

### miRNAS in Proso Millet

Wu et al. (2012) have done preliminary work in characterizing proso millet microRNAs (miRNAs), which are non-coding RNAs important in post-transciptional regulation. Since there are limited proso millet genome sequences, Wu et al. (2012) used ESTs to predict miRNAs instead of using genomic survey sequence analysis as has been done in other crops. Wu et al. (2012) identified 43 potential miRNAs and their gene targets involved in biological processes such as development, metabolism and stress response. They selected 12 miRNAs to validate and eight were verified by Northern blot hybridizations. Their investigations in the role of miRNAs may help understand mechanisms for drought resistance used by proso millet, especially in the dryland farming systems of the PNW.

### SNP Markers, Genetic Linkage Map and Next-Generation Sequencing

Rajput et al. (2016) constructed the first genetic linkage map of proso millet using SNP markers discovered through genotype-by-sequencing (GBS), an application of next-generation sequencing (NGS) protocols (He et al., 2014). Initially, 69,981 SNPs with a minor allele frequency of >0.05 were identified from raw DNA sequence reads. After four levels of filtering, 833 SNPs were eventually used to construct the linkage map which has 18 linkage groups. Since this is the first genetic map for proso millet, each linkage group was considered a chromosome as the haploid genome of proso millet has 18 chromosomes. Using these SNP markers, Rajput et al. (2016) mapped 18 quantitative trait loci (QTL) for eight traits, namely lodging, heading date, plant height, peduncle length, panicle length, grain shattering, 100 grain weight, and grains per panicle. These QTLs accounted for medium to high phenotypic variance (13–35%). After confirmation and validation of these QTLs, the flanking SNP markers could be converted to PCR-based markers and employed in marker-assisted selection (MAS), especially for traits with high additive value such as lodging tolerance, grain shattering tolerance, and number of grains per panicle. The QTLs for these traits explain 22–35% of the phenotypic variance.

### Transcriptome Analysis for Gene Discovery

Using the Illumina high-throughput, paired-end RNA sequencing technology, Yue et al. (2016a) assembled and characterized *de novo* the proso millet transcriptome, the entire collection of RNA sequences in a cell. They used two cultivars for the study, ‘Yumi 2’ which is a waxy, drought-sensitive cultivar, and ‘Yumi 3’, a non-waxy, drought and salt tolerant cultivar. The quality of the raw reads were checked using SeqPrep and Sickle software, and then mixed and assembled using the Trinity program. Four databases were queried to predict the function of the unigenes, namely NCBI No-redundant protein database (NR), Swiss-Prot protein database (Swiss-Prot), Cluster of Orthologous Groups database (COG), and the Kyoto Encyclopedia of Genes and Genomes pathway database (KEGG). Three close relatives of proso millet, *Panicum halli*, *Panicum virgatum*, and *Setaria italica*, were used to predict the function of homologous genes of *P. miliaceum*. The MISA and SOAPsnp softwares were used to detect potential SSR and SNP markers, respectively. Yue et al. (2016a) identified differentially expressed genes (DEGs) between the two cultivars, and performed qRT-PCR analysis after the two cultivars were exposed to low temperature, heat and salt treatments.

RNA obtained from leaves, stem, root, shoots, flower and spike of the two cultivars were equally pooled and sequenced separately, then assembled. The assembly had high accuracy since Yue et al. (2016a) were able to map back to the contigs 93.80% of the reads belonging to ‘Yumi 2’, and 93.29% to ‘Yumi 3’. Of the 113,643 unigenes generated by the Trinity software, 60,352 unigenes were annotated in the NR, Swiss-Prot, COG, KEGG and Gene Ontology (GO) databases. Furthermore, 62,543 unigenes with homologs in the NR databases were annotated to major GO classes, namely cellular component (42.47%), biological process (38.93%), and molecular function (18.60%). To classify their putative function, 33,671 unigenes were aligned to the KOG database and further classified into 25 different functional classes which could be grouped into the ‘function prediction only class,’ ‘signal transduction mechanisms,’ ‘post-translation modification, protein turnover and chaperones,’‘transcription,’ ‘extracellular structures,’ and ‘cell motility.’ In addition, KEGG analysis was used to map 15,514 unigenes to 202 KEGG pathways, namely metabolic pathways, biosynthesis of secondary metabolites, biosynthesis of amino acids, pyrimidine metabolism, purine metabolism, peroxisome, spliceosome and plant-pathogen interaction.

Four differentially expressed genes (DEGs) which may be involved in abiotic stress response were selected for qRT-PCR analysis and their expression profiles examined further under different stress treatments. The *Unigene 34608* is predicted to encode heat-shock factor-binding protein (HSBP1) which can affect HSF1 DNA binding activity and can also negatively regulate response to heat stress. The transcript levels of *Unigene 34608* in ‘Yumi 2’ had small changes in expression levels under cold stress, reduced expression level by 0.26-fold under heat stress, and 0.16-fold under salt stress compared to control plants. In contrast, the expression level of *Unigene 34608* in ‘Yumi 3’ was temporarily elevated under cold and heat stress, even increasing 400-fold under cold stress for 6 h compared to control plants. Its expression declined under salt stress for 24 h. Using the same analysis, the *Unigene 41558* is postulated to encode a CBL-interacting protein kinase 9 (CIPK9) which interacts with calcium sensor and could play a role in low-K^+^ stress. The *Unigene 33484* could play a role in osmoregulation, while Unigene *35973* could encode a zinc-finger protein gene *ISAP1*, which was reported to be involved in regulating cold, dehydration and salt tolerance in transgenic tobacco (Yue et al., 2016a). Further analysis of the proso millet transcriptome using computational prediction identified 32 PmWRKY genes involved in abiotic stress response. In plants, the WRKY genes are key transcription factors regulating various physiological processes, including plant growth, development, and stress response (Yue et al., 2016b). In addition to detecting differentially expressed genes, Yue et al. (2016a) also identified 35,000 SSR and 406,000 SNP loci which can be developed as molecular markers.

### Breeding Proso Millet for Resistance to Biotic and Abiotic Stress

For the PNW, aside from integrating existing millet cultivars into crop rotation systems, there is also a need to breed for cultivars adapted to the region and resilient to biotic and abiotic stresses. Genetic improvement through plant breeding requires effective utilization of diverse germplasm (Kole et al., 2015; Saha et al., 2016), identification and evaluation of core and mini-core collections (Salini et al., 2010; Upadhyaya et al., 2010, 2011; Goron and Raizada, 2015) and accurate phenotyping systems (Lata, 2015). Some traits which can be selected for under PNW growing conditions may include drought tolerance which can influence harvest-index, yield, and water use efficiency (Seghatoleslami et al., 2008); fermentation efficiency (Rose and Santra, 2013); and yield under abiotic and biotic stresses such as low input, salinity, drought, pests, and diseases (Goron and Raizada, 2015).

Cultivar development and genetic improvement of proso millet, as in other small millets, has been largely achieved through the direct selection of promising germplasm (Naylor et al., 2004). In the U.S., there are 15 cultivars of proso millet available to growers. Nine of these cultivars were selections from adapted landraces, and six were developed through hybridization followed by traditional breeding (Rajput et al., 2016). The variety ‘Dawn’ was the first of the modern proso varieties, and is the parent of most of the cultivars released in Nebraska (Lyon et al., 2008a). The white-seeded varieties ‘Sunrise,’ ‘Huntsman,’ and ‘Earlybird’ were developed from crosses, with the F_1_ and F_2_ seed increased in the greenhouse, and the F_4_ selections included in yield nurseries and regional trials (Baltensperger et al., 1995b,c, 1997). Prior to the elucidation of the molecular basis of the waxy endosperm trait by Hunt et al. (2010, 2012), and development of primer-based markers for the *Waxy* gene by Araki et al. (2012), the trait was investigated using segregation analysis of F_3_ populations. These F_3_ populations were derived from crosses between wild-type accessions such as ‘Earlybird,’ ‘Sunrise’ and ‘Huntsman’ and waxy accessions obtained from the USDA-ARS proso collection. It was postulated that a duplication of the inheritance factor of the recessive waxy trait occurred. The double mutant waxy lines resulted from the outcrossing of single null lines which arose from independent mutations in different backgrounds (Graybosch and Baltensperger, 2009). These double mutants were then selected and perpetuated in ancient Asia due to preference for glutinous texture. The first waxy proso millet cultivar released in the U.S. is ‘Plateau,’ with ‘Huntsman’ as the female parent and PI 436626 as the donor parent of the waxy trait (Santra et al., 2015).

Proso millet has few significant diseases in the Great Plains and Colorado, with head smut *Sporisorium destruens* (Schltdl.) Vanky = *Sphacelotheca destruens* (Schltdl.), bacterial stripe disease (*Pseudomonas avenae*), and kernel smut (*Ustilago crameri*) being the more important ones (McDonald et al., 2003; Lyon et al., 2008a) though there are no reports in the U.S. of molecular interventions to manage these diseases. In Ukraine, there have been efforts in breeding *P. miliaceum* for resistance to head smut and melanosis, which is blackening of the grain under the husk attributed to bacterial pathogens *Pseudomonas syrinagae* and *Xanthomonas campestris* pv. *holcicola.* Smut resistant varieties have been released in 1986 (Kh86) and 1989 (Kh22) by the Institute of Plant Production, Breeding and Genetics in Khar’kov, with some mutant lines having only 1.8 – 8.3% infection under greenhouse conditions (Konstantinov et al., 1991). In China, Zhou et al. (2016) found moderate genetic diversity among 51 isolates of *Sporisorium destruens* using RAPD markers. The smut isolates also varied in virulence and were grouped into three pathotypes. These isolates were used to screen 280 accessions for resistance, and 10 accessions were found to be potential differential hosts for identification of pathotypes. The study of Zhou et al. (2016) laid the groundwork for breeding for head smut resistance of proso millet in China.

There are no serious insect pests for proso millet in the Great Plains, Colorado (McDonald et al., 2003; Lyon et al., 2008a) and elsewhere in the U.S. However, several insect pests still pose a constraint to proso millet production, and at present, resistant cultivars are developed through traditional breeding. The early proso cultivars released in the U.S. such as ‘Huntsman,’ ‘Earlybird’ and ‘Sunrise’ are resistant to the Russian wheat aphid [*Diuraphis noxia* (Mordvilko)] (Baltensperger et al., 1995b,c, 1997). In Oklahoma, Wilson and Burton (1980) screened proso millet cultivars for oviposition and feeding of five insect pests of grains, specifically cinch bugs [*Blissus leucopterus leucopterus* (Say)], corn earworms [*Heliotis zea* (Bodie)], fall armyworms [*Spodoptera frugiperda* (J.E. Smith)], southwestern corn borers [*Diatraea grandiosella* (Dyar)] and yellow sugarcane aphid [*Sipha flava* (Forbes)]. Corn earworms and armyworms deposited readily on proso but southwestern corn borers did not. Moreover, corn earworms larvae preferred the cultivar ‘Dawn.’ Meanwhile, 153 proso lines from the North Central Plant Introduction Station were evaluated for resistance to fall armyworm, with eight lines reported as resistant (Wilson and Courteau, 1984). On the other hand, weeds are managed using cultural practices such as crop rotations, tillage, row spacing and plant populations, and the use of pre-and post-emergence herbicides (McDonald et al., 2003; Lyon et al., 2008a).

Since most areas of the PNW fall under dryland farming systems, it is important to breed for drought and salinity tolerance in proso millet. Drought reduces the productivity of crops by limiting the water available for metabolic processes, but the level of reduction varies since drought interacts with factors such as genotype, developmental stage of the crop when drought occurs, as well as the duration and severity of the drought (Ngara and Ndimba, 2014). The effect of drought at the vegetative, ear emergence and seed filling stage on five proso millet genotypes was investigated by Seghatoleslami et al. (2008). Their results show that grain yield was reduced for all genotypes when drought occurred at the ear emergence stage, though two genotypes had higher harvest indices than the rest. Mid-season water stress was imposed on proso millet genotypes belonging to different maturity groups (early, middle, and late maturing) when 50% of the plants were at the flowering stage. The water stress was continued for 10 consecutive days, followed by re-irrigation to field capacity until harvest (Emendack et al., 2011). Compared to *Sorghum bicolor*, *P. miliaceum* was more susceptible to water stress, with 77% reduction in yield, particularly with the middle and late-maturing types (Emendack et al., 2011). In another study, *P. miliaceum* had 36% yield reduction when water deficit was imposed both at pre- and post-heading stages, due to smaller number of grains per panicle, fewer panicles and reduction in total dry weight (Matsuura et al., 2012).

A primary abiotic stress, such as drought or salinity, can produce secondary stresses such as osmotic and oxidative stress (Wang et al., 2003). Drought and other abiotic stresses can cause early aging in plants, often observed as leaf senescence. In response, a series of defense reactions are initiated, physiological and biochemical reactions which can be used as indexes for drought resistance. Superoxide dismutase (SOD), catalase (CAT), and peroxidase (POD) are antioxidative enzymes involved in plant defense against drought (Zhang et al., 2012a). Karyudi and Fletcher (2002) found that of the 14 accessions of *P. miliaceum* tested, those with higher osmoregulative capacity had some degree of drought tolerance. Osmoregulative capacity was determined from the relationship between osmotic potential and leaf water potential on flag leaves of plants at heading stage. Jia et al. (2008) simulated drought stress conditions with PEG-6000 and investigated the biochemical characteristics of seedlings of two cultivars ‘Yumi 1’ and ‘Yumi 3’ under greenhouse conditions. They noted differences between the two cultivars, with the more susceptible cultivar ‘Yumi 1’ with increased electrolyte leakage, malondialdehyde content (MDA), praline and soluble surge content, while the there was reduced activity of the POD and SOD under 10–30% PEG stress for 8 days. Using the same simulated drought conditions, Zhang et al. (2012b) subjected 10 proso millet cultivars to simulated water stress (0.25 g/ml PEG-6000) and found that SOD and POD activity and chlorophyll content can be used as effective indicators of drought resistance in proso millet during the seedling stage.

Leaf senescence and antioxidant enzymes in three cultivars of proso millet after anthesis were studied by Zhang et al. (2012a). The changes in chlorophyll content, antioxidant enzymes (SOD, CAT, POD), MAD and superoxide anion during seed filling to maturity were investigated with the primary goal of using these indices in the selection of drought resistant varieties. The cultivar ‘Ningmi 13’ had slower degradation ratio of chlorophyll content, higher activity of SOD and CAT, lesser POD, and smaller accumulation of MDA and superoxide anion, resulting to delayed leaf senescence and prolonged leaf functional period. Therefore, longer functional leaf period and higher SOD activities can be used as indices for selection of drought tolerant genotypes (Dai et al., 2011; Zhang et al., 2012b).

In plants, abiotic stress induces genes which encode proteins to protect plant cells, and genes which encode for proteins that regulate gene expression and signal transduction, such as transcription factors and protein kinases (Ngara and Ndimba, 2014). To investigate the genes induced by drought in *P. miliaceum*, Lin et al. (2006b) used a forward subtracted cDNA library constructed from normally watered leaves and leaves rehydrated after drought. They employed a suppressive subtraction hybridization technique to construct the cDNA library and 60 positive clones identified and sequenced. Of the 60 sequences, 32 EST were found highly homologous to known plant sequences expressed in response to abiotic or biotic stress. Furthermore, 28 ESTs are homologous to known proteins involved in signal transduction, transcription and protein processing. From this cDNA library, AFLP markers were generated by Lin et al. (2006a) to analyze genes differentially expressed in seedlings watered normally, those subjected to drought, and seedlings rehydrated after drought. Twelve fragments were amplified from the leaf samples under drought or rehydration regimes, then cloned and sequenced. Sequence analysis showed that one fragment was similar to UDP-*N*-acetylglucosamine-*N*-acetylmuramyl-(Pentapeptide), two fragments had significant homologous protein sequence with a rice retrotransposon, while two other fragments had significant homologous protein sequence with two hypothetical proteins (Lin et al., 2006a). Using the same cDNA library, Lin et al. (2008) amplified a full-length cDNA of *S*-adenosylmethionine synthase (SAMS) gene using PCR. The expression pattern of this gene was studied using semi-quantitative RT-PCR, and results show that its expression declined under drought, increased after rehydration, and then settled to normal levels 6 h after rehydration. It is postulated that this gene plays a key role in drought tolerance and water use efficiency (Lin et al., 2008).

### Prospects for Future Genomic Research

Molecular markers based on coding and non-coding regions of the proso millet genome were developed and used in genetic diversity analysis of landraces, breeding lines and cultivars. These molecular markers were also used on phylogenetic and phylogeographic studies to elucidate the genetic relationships of accessions and their geographical origins. In addition, molecular markers were used to trace the spread of proso millet from its center of domestication, and identify its wild progenitor. However, in relation to its abundant morphological variations, the genetic diversity of proso millet has not yet been adequately assessed (Trivedi et al., 2015; Liu et al., 2016). As in other millets, there are no mutants or mutant populations in proso millet to study gene functions through reverse genetics (Saha et al., 2016); the waxy mutants are natural variants of the *waxy* gene (Hunt et al., 2010). Previously, inadequate molecular markers combined with the challenges of a tetraploid genome such as inconsistent meiotic processes, allelic and non-allelic combinations, and poor correlation between genotype and phenotype (Saha et al., 2016) have made it difficult to conduct genetic and genomic studies in proso millet. However, with the discovery of SNPs by GBS (Rajput et al., 2016) and the identification of differentially expressed genes and thousands of SSR and SNP loci by transcriptome analysis (Yue et al., 2016a,b), a considerable number of molecular markers are currently available for genomic research in proso millet.

With the construction of the first genetic linkage map using SNPs and the first QTL mapping study conducted in proso millet (Rajput et al., 2016), there are now promising tools for molecular breeding of this crop (Varshney et al., 2010). With the discovery of thousands of unigenes, SSR and SNP loci by transcriptome analysis (Yue et al., 2016a,b), genetic linkage analysis, genome-wide association studies and genomic selection in proso millet are now distinct possibilities. Marker-assisted selection can also be incorporated into proso millet breeding programs once the SNPs flanking QTLs have been confirmed and validated (Rajput et al., 2016). These molecular tools can be used to further study the rich genetic diversity of the proso millet accessions preserved in genebanks worldwide, in addition to the landraces grown and preserved by farmers which have helped agrarian communities survive for generations in marginal lands (Newmaster et al., 2013). These landraces will continue to be a rich source of unique alleles for breeding and genetic improvement of proso millet (Goron and Raizada, 2015).

## Conclusion

Proso millet possesses many unique characteristics that make it a promising rotational crop for the PNW region of the U.S. Proso millet can utilize moisture more efficiently than wheat and long-season crops such as corn, grain sorghum, or sunflower because it has one of the lowest water requirements of any grain crop. Proso millet could help improve wheat productivity through its capacity to control winter annual grassy weeds, reduce insect and disease pressure, and preserve deep soil moisture for the subsequent wheat crop. In addition, proso millet can provide a rotational benefit to the dryland farming of the Palouse region of Washington, Oregon, and Idaho, where wheat is the keystone crop. Proso millet cultivation could promote diversification of wheat-based cropping systems and provide a regionally available source of a highly nutritious cereal grain.

## Author Contributions

CH, JM, JDG, GG, and KM conceived and wrote the manuscript. MR, KK edited and provided additional information. All authors read and approved the final manuscript.

## Conflict of Interest Statement

The authors declare that the research was conducted in the absence of any commercial or financial relationships that could be construed as a potential conflict of interest.

## Footnotes

1 http://www.agmrc.org/commodities-products/grains-oilseeds/proso-millet/

2 http://www.rma.usda.gov/pilots/feasible/pdf/millet.pdf

3 http://cses.washington.edu/cig/pnwc/pnwc.shtm

4 http://rainfall.weatherdb.com/l/19858/PaloUSe-Washington

5 http://extensionpublications.unl.edu/assets/pdf/g2218.pdf

6 https://www.colorado.gov/pacific/agmarkets/millet

## Abbreviations

ATP: Adenosine triphosphate
BP: before present time
CDA: Colorado Department of Agriculture
CVD: cardiovascular disease
GDD: Growing Degree Day
PNW: Pacific Northwest
WSU: Washington State University
